# An investigation into the use of Full-body Low Dose X-rays Imaging system in South Africa: Radiographer's perspective

**DOI:** 10.1016/j.afjem.2023.05.009

**Published:** 2023-06-12

**Authors:** Muchui Julius Thambura

**Affiliations:** Department of Radiography, Faculty of Health Sciences, University of Pretoria, Pretoria, South Africa

**Keywords:** Lodox, Ionising radiation, Trauma imaging, Conventional x-ray system, patient referral, emergency unit, image quality

## Abstract

**Introduction:**

A low dose x-rays alias Lodox© statscan was originally developed in South Africa to detect smuggled diamonds in the mines. Later, hospital trauma units began to use it as a screening tool for gross pathology on trauma patients. This imaging system became popular because of its use of low radiation doses and its ability to perform anterior posterior (head to toe image) image in under 13 seconds. Anecdotal evidence confirms that patients were referred for additional regional images on conventional x-ray systems after Lodox imaging. Thus, patients were subjected to additional ionising radiation, long waiting times as well as additional charges for secondary radiological examinations.

**Objective:**

This research aimed at investigating the extent to which Lodox was used in trauma units (n=28) in South Africa.

**Method:**

In this descriptive cross-sectional research. researcher invited one radiographer from each of the 28 hospitals in South Africa that were using Lodox.

**Results:**

Out of twenty radiographers who responded, it was found that most hospitals were referring patients for additional conventional x-ray images (Figure 1); for example, for chest x-rays. This was done despite the patient having undergone radiological procedures and examinations by the Lodox imaging system that was similar to those performed by conventional x-ray systems.

**Conclusion:**

Lodox was used for a successful diagnosis Thus, researcher recommends an imaging protocol for Lodox to be developed for guiding the referral of patients after the Lodox scanning has been performed.

## African relevance

• The Lodox imaging system is currently used at trauma units in some African countries like South Africa and Nigeria. It has also been adopted for use at trauma units in Europe and United States of America.
• The use of this imaging system saves time on management of trauma patients with multiple injuries.
• In addition to this, this imaging system is cost effective. There is no need for room modifications as required by radiation control directorates/ boards of various country. The radiation used is five times less that that used in conventional x-rays.
• This imaging system will be of added value in Africa where we are resource-constrained, and healthcare systems are underfunded.
• The Lodox images may be used where there are no x-rays units. The images produced are of high diagnostic value. For example, the visualisation of full spine as well as chest abdomen and pelvis is possible in one image.
• Most Lodox imaging systems are installed in Africa. This research proposes a justification for no additional imaging after a patient has undergone a Lodox scan. The reduced retake of the images will lower radiological examination bills, lower ionising radiation doses and reduce the patient waiting time at both radiology departments and trauma units. This will also reduce the workload on the staff at radiology department while improving the quality of care and expediting healing process of the patient.

## Introduction

Developed in the early 1990s, the low dose x-rays (Lodox© Statscan or Lodox) was initially designed as an industrial tool for screening for smuggled diamonds in the diamond mines [1]. Due to its low ionising radiation, imaging speed and ability to produce a full body image in 13 seconds, the Lodox was adopted as an adjunct screening tool in trauma and emergency units for detecting foreign bodies and gross pathologies[2]. Despite producing images of superior quality, trauma personnel were requesting additional examinations using conventional x-ray system even after the patient had undergone a Lodox scan. Requesting secondary images meant that patients were exposed to additional ionising radiation. Requesting additional imaging defeats the major benefit of the use of Lodox which uses a low radiation dose to produce radiological images [2]. In this research, the researcher investigated the extent to which patients, who had undergone full-body Lodox scanning, were referred for additional conventional x-rays.

## Method

### Research design, sample, recruitment, and data collection

This was a descriptive cross-sectional study [3]. The researcher purposively extended an invitation to radiographers who had used Lodox in the 28 hospitals over a long period [4]. Ethical approval was obtained from the University of Pretoria Research Ethics Committee under number 486/2017. The ethics committees in the 28 hospitals also approved this research before data collection. All respondents signed informed consent forms before completing an online questionnaire. Confidentiality was maintained by withholding details of both the hospitals and the respondents [5].

## Data analysis

Descriptive statistics were used to report frequencies and proportions for both demographic and non-demographic variables. Data was analysed using the Statistical Analysis System (SAS Version 9.4, released by SAS Institute Cary, North Carolina in July 2013).

## Results

**Figure 1:**
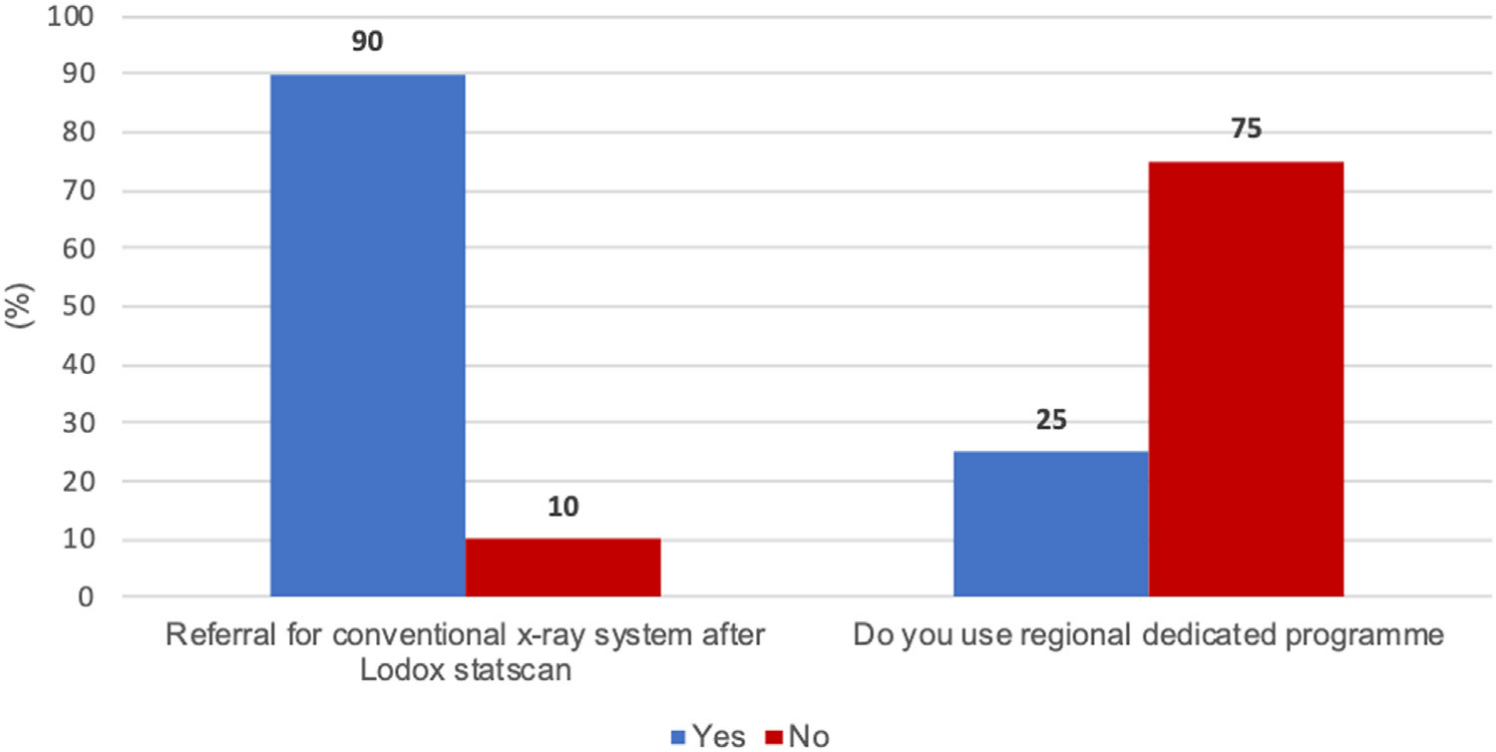
Patient referral for conventional x-ray imaging and the use of regional dedicated programme of Lodox.

Of the 28 radiographers, 20 (71.4%, n=20 responded to the survey. Figure 1 shows the rates of referral of patients for conventional x-rays after a Lodox scan had been performed and the rate of use of the regional dedicated programme for Lodox scanning at the 20 hospitals.

Among the 20 hospitals that responded, eighteen (90.00%, n=20), referred patients for conventional x-ray imaging after they had undergone Lodox imaging. Most of the hospitals (15; i.e., 75%, n=20) did not use the regional dedicated programmes meant for specified regional imaging on the Lodox system (figure 1).

**Figure 2:**
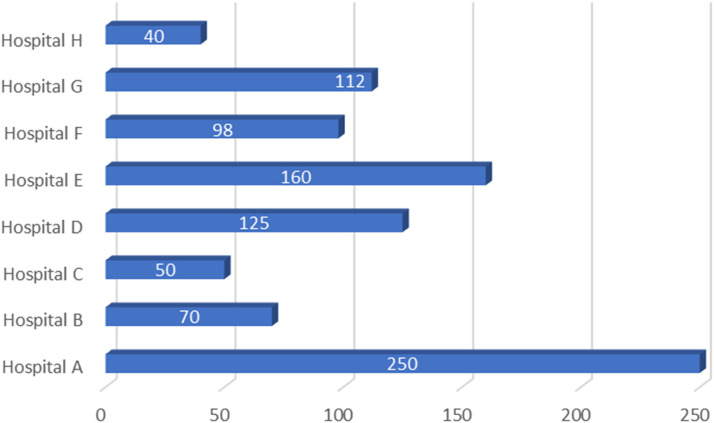
Average number of patients undergoing Lodox imaging in a month.

Figure 2 shows a monthly statistics of patients that underwent a Lodox scan in various hospitals.

Hospital A had the highest number(250) of the patients undergoing Lodox scanning per month while Hospital H had the least number(40). On average, the eight hospitals were screening 113 patients monthly.

**Figure 3:**
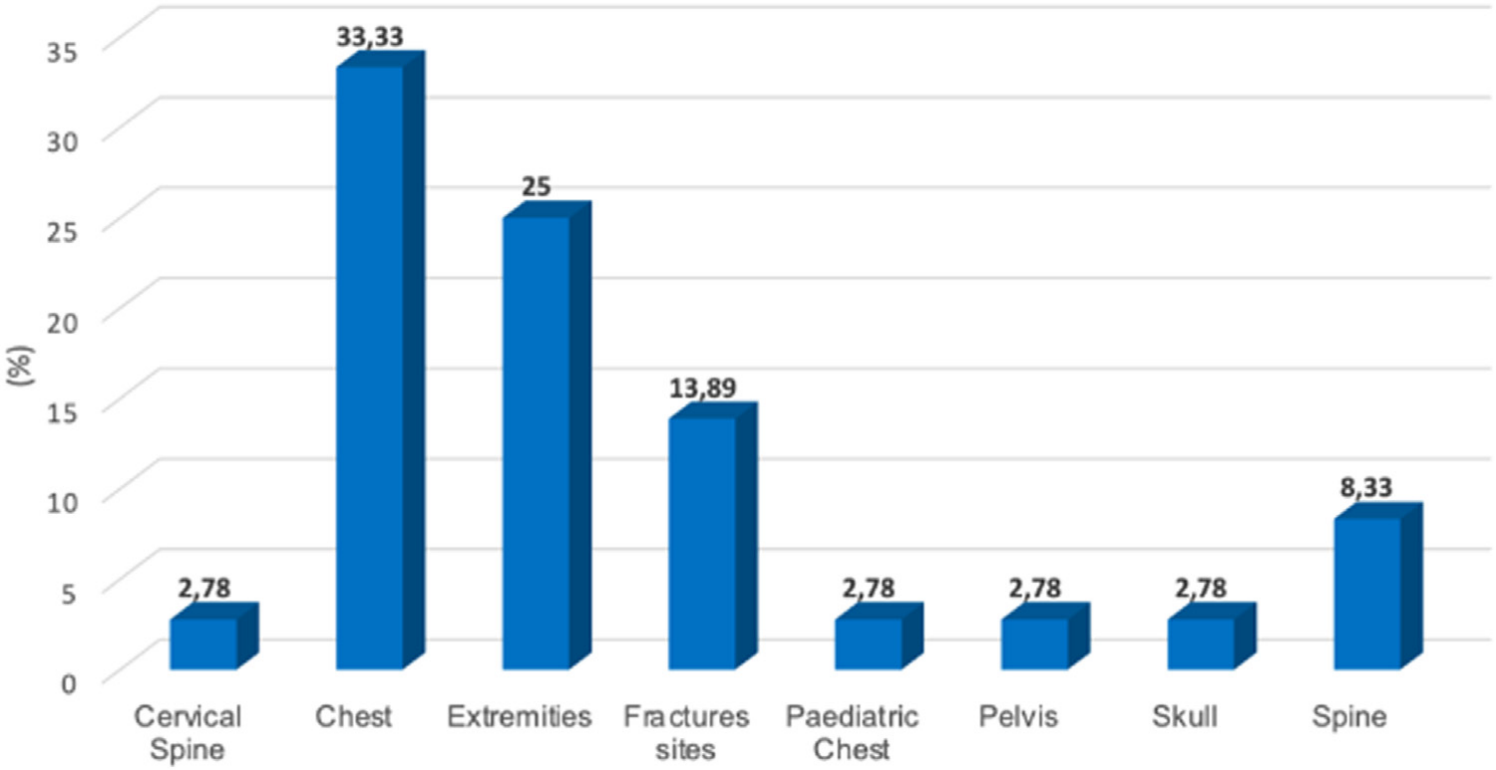
Number and types of radiological examinations that are referred for conventional x-rays after Lodox scanning.

Figure 3 displays the number of various types of examination that were referred for conventional x-ray imaging after a Lodox scan had been performed.

Figure 3 shows that chest x-rays (adult and paediatric chest) were the most requested examination. Extremities (25.00%) and fractured sites were also referred for a repeat x-ray using the conventional x-ray system. Skulls, cervical spine and pelvis were the least requested

**Figure 4:**
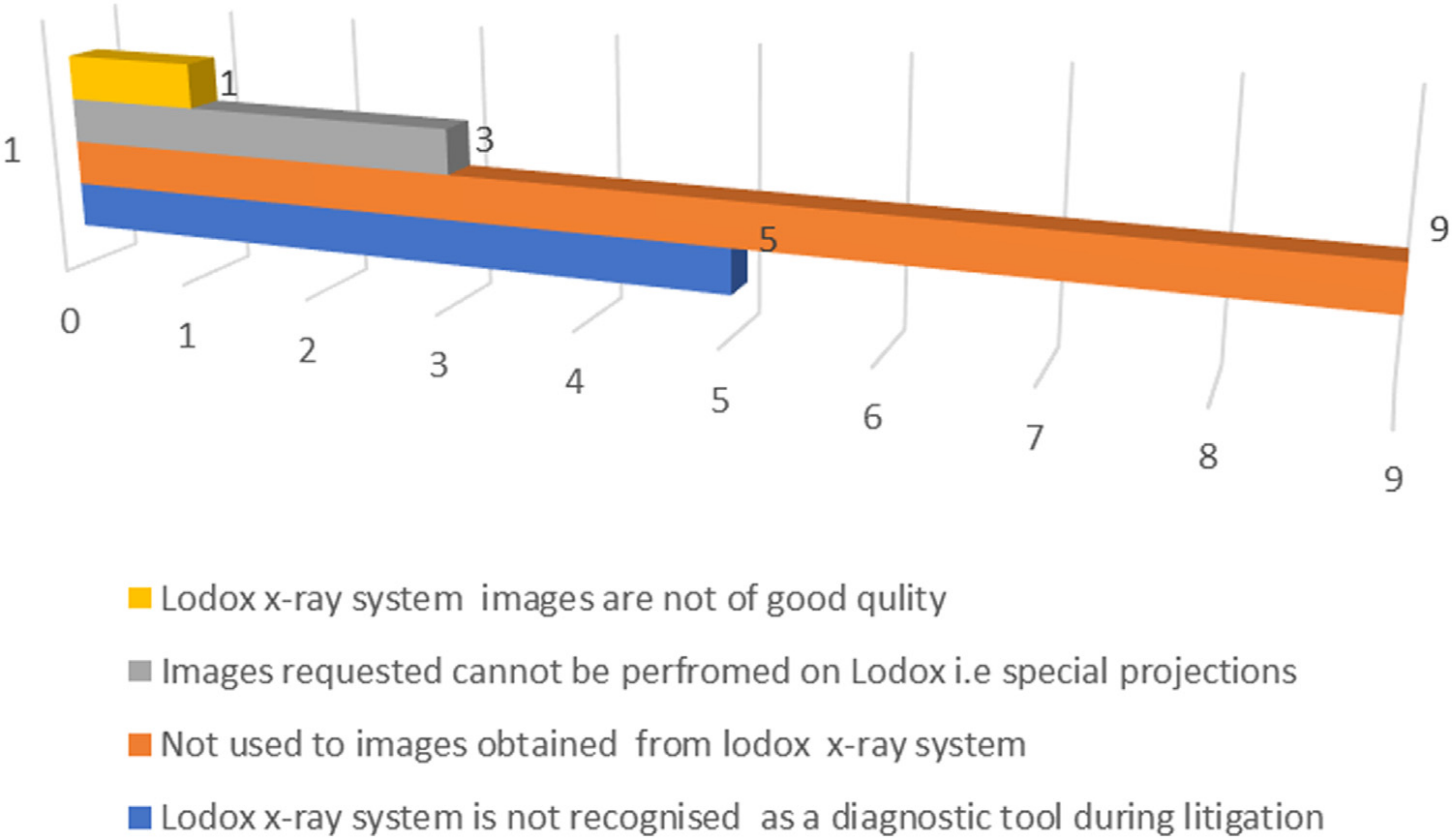
Reasons for referring patient for conventional x-rays after Lodox imaging.

Figure 4 shows reasons conventional x-ray images were requested after Lodox imaging had been performed.

Among the 20 respondents, 10 (50.00%) indicated that the referring physicians were not used to Lodox x-rays images; five (33.37%) indicated that Lodox images were not recognised as a diagnostic tool in case of litigation; three (16.67%) indicated that images that were requested for conventional x-ray system could not be performed successfully using Lodox (for example, some special projections) and the other two of the 18 respondents indicated that Lodox images were not of good quality.

**Figure 5:**
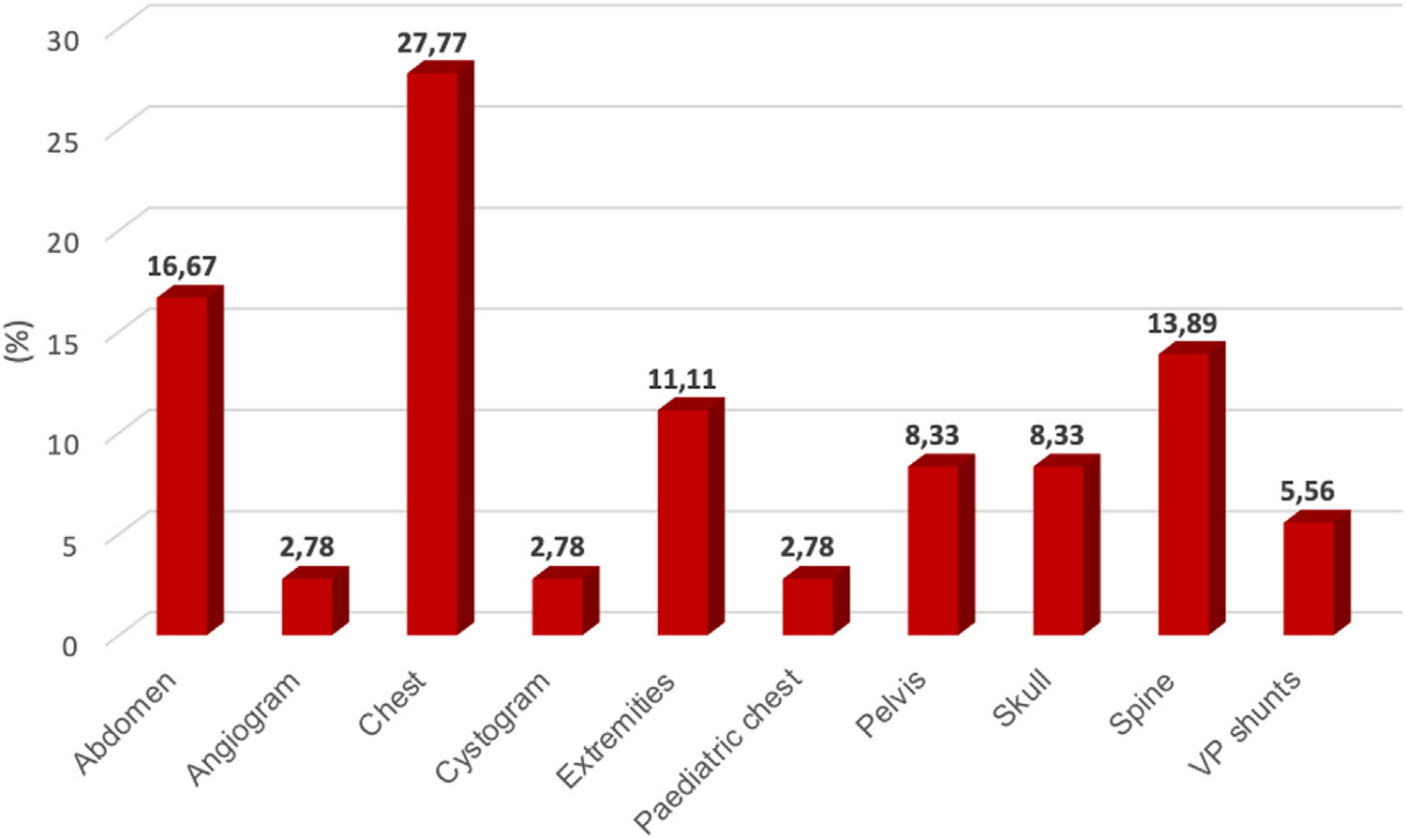
Non-trauma radiological examinations that are performed using Lodox.

Figure 5 indicates the various imaging examinations performed at South African hospitals using Lodox.

Out of the 36 images that were retrieved from the imaging system, chest images had the highest number with 11 images (30.55%, n=36) out of the 36. Chest images comprised of adult and paediatric chest examinations; abdominal images numbered 16 (16.67%, n=36); spinal images numbered five (13.89%, n=36), extremities numbered four (11.11%, n=36) while both pelvis and skull images had three (8.33%, n=36) images each. The Lodox was also used for two fluoroscopic procedures, which included angiography (2.78%, n=36) and cystography(2.78%, n=36.

**Figure 6:**
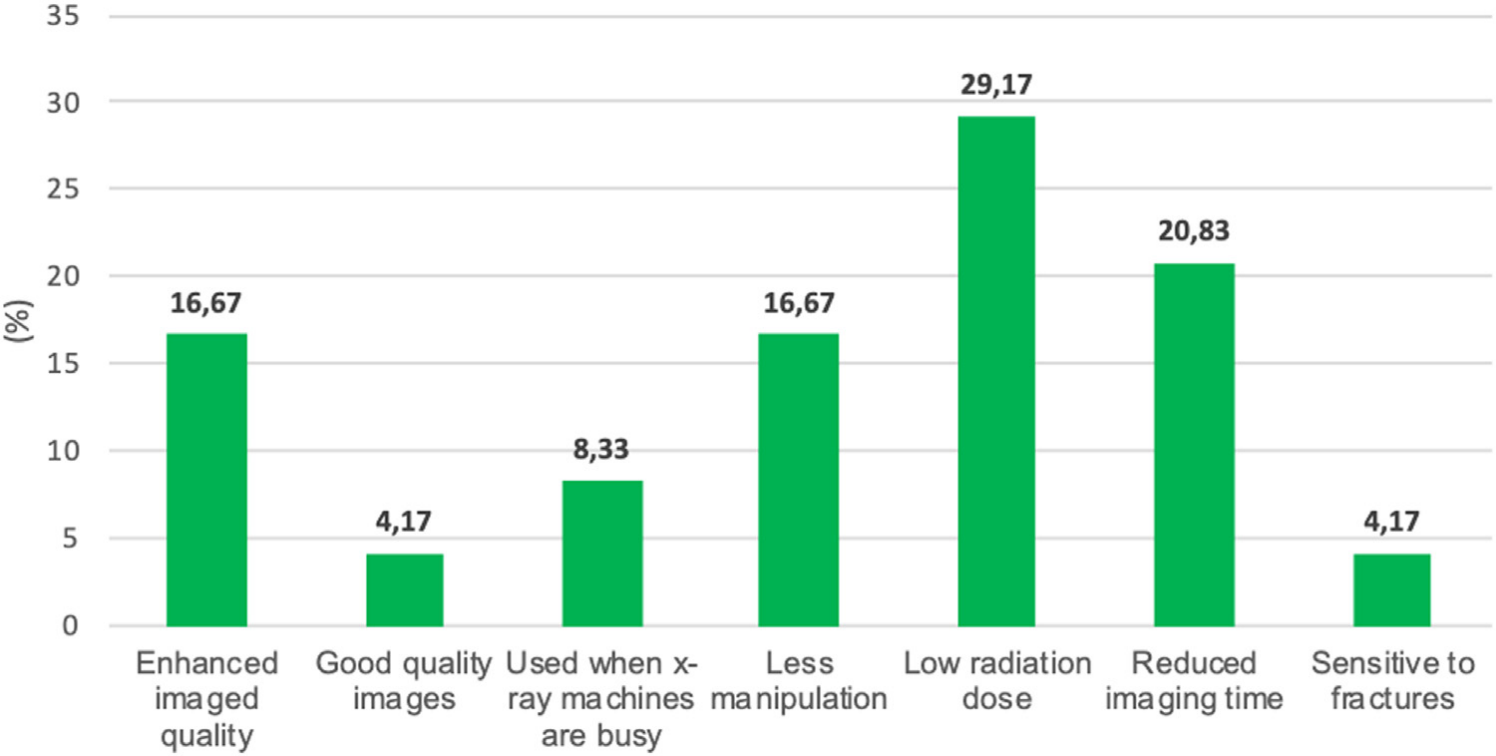
Reasons for using Lodox imaging system for non-trauma examination.

Figure 6 displays radiographers ’opinions on use the Lodox imaging system for non-trauma imaging examinations.

The researcher inquired why radiographers were using Lodox for non-trauma examinations. Twenty-four respondents answered this question. Low radiation dose was the major reason for using Lodox. This appeared seven times (29.17%) among the 24 responses given. Reduced imaging time when Lodox was used appeared five times (20.83%, 5/24). Lodox needed less manipulation of the patient and enhanced image quality of the Lodox appeared four (16.67%, 4/24) times. The Lodox x-ray system was used when other imaging equipment was not available or non-functional appeared twice (8.33%). Lodox being sensitive to fractures and producing images of better quality than the conventional x-ray system was indicated by one (4.17%,1/24) respondent.

## Discussion

In this study, the researcher enquired about the extent to which patients were referred for additional conventional x-ray imaging following a full-body Lodox scan. In most hospitals (90%), trauma doctors and nurses sent patients for additional x-ray imaging of the chest (Figure 1). It also appears that Lodox imaging is a popular radiological examination (Figure 2) to the extent that some of the hospitals had up to 250 patients undergoing Lodox imaging monthly.

Sending patients for additional x-ray imaging after Lodox imaging defeats the intended purpose of using “low dose x-ray imaging” which is a key characteristic of the Lodox x-ray system [1,6]. Ionising radiation is carcinogenic and excessive exposure is associated with genetic aberration and subsequently genetic mutations [7,8]. Overall, repetitive exposure to ionising radiation increases the chances of stochastic and deterministic effects of ionising radiation [8,9]. Because of the dangers associated with ionising radiation, both the European Commission and the International Atomic Energy Agency have emphasised that requests for radiological examinations should be justified, and the use of ionising radiation should be optimised. However, repeat radiological examinations are still being requested [10]. Patients undergoing both Lodox and conventional x-ray imaging are exposed to almost double the radiation dose than when only one of the two systems is performed [11,12]. On the other hand, additional examinations may also influence the patient's hospital bill and lengthen the duration of investigations. In public healthcare systems, over-servicing adds costs to the already expensive healthcare services in South Africa [13].

Additional x-ray imaging using conventional x-ray system were mostly requested for chest conditions, both adult and paediatric. At trauma units, clinical signs, physical examination, vital signs and oxygen saturation can identify high-risk patients without necessarily sending the patients for chest x-ray imaging [14]. As argued by Daya et al., Lodox scans are known to produce superior images to conventional x-ray images, including the diagnosis of pneumothorax lung contusions and mediastinal injuries such as a ruptured aorta, pneumomediastinum, thoracic skeletal fractures and peripheral bone lesions [11]. Daya et al. [15] also emphasised that Lodox images are effective in diagnosing chest pathologies in paediatric patients.

Among the reasons why patients were referred for secondary images using the conventional x-ray system were that physicians were not used to Lodox images(50.00%, n =18). Since its discovery in 1999, the use of Lodox has been limited to screening patient at the trauma unit as an adjunct imaging tool.[117] Despite its adoption, not all the hospitals in South Africa have installed this imaging system. Thus, most healthcare personnel have limited exposure to the use of this imaging system. Five (27.78%) of the participants indicated that Lodox images were not recognised as diagnostic images for litigation. Although Lodox was approved for radiographic examination by the US Food and Drug Administration (FDA) in 2002, there are no publications on its recognition for use as evidence to rule out a pathology in a court of law [15]. Therefore, its advancement in forensic imaging investigations is paramount. Three (16.67%) of the respondents indicated that images that were usually requested could only be performed using a conventional x-ray system because radiographic images of some of the regions of the body require angulations of x-ray beam in a specified direction [8]. The ability of an imaging system to be angled in various directions is advantageous more so on trauma patients who requires minimal manipulation. These different angles enable various projections to be performed hence visualisation of the pathologies in different dimensions [16]. Conventional x-ray systems can be adjusted to various source image distances (SID) as well as different angles while the Lodox can only be rotated axially up to 90^o^ [8,15]. The ability of the conventional x-ray system to be adjusted to different angles at different source image distance(SID) minimises patient manipulation that may trigger pain or severe injuries to the patient [8,17]. This could be the reason why patients are referred for conventional x-rays after Lodox images have been obtained. One (5.56%) respondent indicated that images from Lodox are of poor quality. This finding contradicts the findings by the Boffard et al. in 2006 that chest, pelvic and cervical spine, cervical thoracic junction on Lodox images were similar to those of the conventional x-ray system [18]. Despite the questions about the inherent qualities of the imaging system, the quality of radiographic images may be determined by radiographic errors when positioning the patient as well as the presence of artefacts on the patient during the imaging procedures [17].

In South Africa, radiographers are using the Lodox system to perform other radiological examinations alongside screening of trauma patients (Figure 5). Previous research suggests that the Lodox system has the potential to replace conventional x-ray systems for routine applications [19,20]. The researcher found that trauma units in SA were using Lodox as a reliable alternative for conventional x-ray imaging to diagnose pathologies of the chest, abdomen, spine and extremities (Figure 5). Previously, the Lodox system had produced useful images of the whole spine, [1] facial fractures [21] and wrist and ankle joint fractures [22]. Furthermore, this research found that Lodox was used to evaluate pathologies of the skull. This is in line with suggestions that were made regarding the use of the Lodox for diagnosing and follow-up procedures on paediatric tuberculosis [15]. The findings from this study, thus, support an extended application of the Lodox system for adult and paediatric imaging.

Interestingly, South African radiographers also reported using Lodox for fluoroscopic procedures such as angiography and cystography (Figure 5). Using the Lodox system for follow-up imaging taken at various intervals to evaluate pathophysiology [19] may significantly reduce radiation exposure for the patient due to its low radiation dose associated with this imaging equipment [1,18]. Noteworthy is that Lodox has previously been used for angiography, where Computed Tomography scanning (CT scan) and fluoroscopic units were unavailable [21]. Additionally, Lodox x-ray imaging may be useful in visualisation of catheter placements to assist healthcare professionals on full-length view and patency of the catheter [19]. It was also reported that the Lodox system allows the visualisation of the full-length of contrast-enhanced blood vessels and the urinary system during fluoroscopic procedures. Visualisation of these organs for intervention or therapeutic purposes is essential for efficient and effective diagnosis of a condition [17]. The researcher inquired why Lodox was preferred for procedures other than routine full-body screening at trauma units. Radiographers indicated that Lodox emits low amounts of ionising radiation compared to conventional x-ray systems (29.17%), which they consider to be beneficial.

## Conclusions

The Lodox system is being used in most trauma units in SA for rapid diagnosis in trauma cases and as a backup when other diagnostic equipment is not available or too busy. South African radiographers using the Lodox system are positive. Despite radiologists not providing radiological reports on emergency Lodox scans, several hospitals are routinely referring all patients for additional conventional x-ray imaging following Lodox scans. This may expose patients to higher doses of radiation, and waiting times in radiology departments may have added risks, complicating their condition. Additionally, requesting additional images must be done in good faith where holistic analysis of non-maleficence and beneficence needs to be balanced to avoid over-servicing and an idiosyncratic outcome. Additional conventional x-ray imaging also increases hospital costs for the patients and added labour for radiographers. The researcher suggests that the diagnostic capacity of Lodox images be further investigated to encourage trauma specialists to use the Lodox system as a diagnostic aid in the trauma room, rather than routinely referring patients for additional conventional x-ray imaging.

## Dissemination of results

The results for this study were presented at the Pan-African Conference of Radiology and Imaging held between 14^th^ -17^th^ January 2019; at the 2^nd^ Cape Town International Trauma Conference in 21^st^ -22^nd^ November 2019; and as a feasibility study for the researcher's PhD defence at the University of Pretoria on 13^th^ November 2018

## Declaration of Competing Interest

The author declares no conflict of interest.
